# X-ray and DNA Damage: Limitations of the Dose as a Parameter for In Vitro Studies

**DOI:** 10.3390/ijms242316643

**Published:** 2023-11-23

**Authors:** Ion Udroiu, Antonella Sgura

**Affiliations:** Department of Sciences, Università Roma Tre, Viale G. Marconi 446, 00146 Rome, Italy; antonella.sgura@uniroma3.it

**Keywords:** DSB, ionising, linear–quadratic, micronuclei, radiobiology, RBE

## Abstract

A century of studies has demonstrated that the magnitude of a radiation dose determines the extent of its biological effect. However, different types of radiation show different levels of effectiveness. Although all types of X-rays are usually considered to be equivalent, several authors have demonstrated an inverse relationship between photon energy and the biological effectiveness of the X-ray. Nonetheless, the differences among 50–100 keV X-rays are usually considered absent. However, comparing different types of X-rays with different energies is not easy since they are often used with different dose rates, and the latter can be a confounding factor. We compared the biological effectiveness of X-rays with different photon energies but with the same dose rate. Moreover, we also studied X-ray with different dose rates but the same photon energy. Biological effectiveness was assessed measuring DNA damage and cell survival. We confirmed that both the dose rate and photon energy influence the effectiveness of an X-ray. Moreover, we observed that differences in the 50–100 keV range are detectable after controlling for dose-rate variations. Our results, confirming those of previous studies in a more consistent way (and accompanied by hypotheses on the importance of the number of incident photons), underline the limitations of using the dose as the sole parameter for in vitro studies.

## 1. Introduction

For about a century, it has been established that the magnitude of a dose of ionising radiation delivered to a biological system determines the magnitude of its effect. In fact, Wigoder and Patten [1] were the first to establish that biological effects (in that case, growth inhibition and mitosis delay) are proportional to the X-ray dose (quantified by them in röntgen, i.e., a measure of exposure).

Successively, it has been discovered that with the same absorbed dose (now measured in Grays, Gy), different types of radiation can cause different levels of biological damage. This led to the concept of relative biological effectiveness (RBE) as the ratio of the effectiveness of one type of radiation relative to another one [2]. Thus, the equivalent dose *H* (measured in Sieverts, Sv) is the product of the absorbed dose (*D*) and the radiation weighting factor (*W_R_*), the latter being defined by the RBE. X- and γ-rays are used as references for the RBE of other types of radiations; thus, they have an *W_R_* equal to 1 [3].

Although not widely known, X-rays with different photon energies (measured in kiloelectronvolts, keV) have different degrees of effectiveness. Indeed, measuring the survival fraction [4], neoplastic transformation [5], and dicentric chromosomes [6,7,8,9,10,11] in cells exposed to different types of X-rays, it was shown that biological effectiveness was inversely proportional to photon energy. We would like to add that as early as the 1920s, it was noted that softer X-rays (with lower energies) have more marked effects than harder X-ray on cell cultures [12,13].

Another issue is related to the dose rate: measuring the survival fraction [14,15], unrepaired DNA double-strand breaks [15], and micronuclei [16], several authors noted that biological effectiveness increases with the dose rate. Also, in this case, century-old studies [17,18] noted that more marked effects are produced when the same dose is given in a shorter time (i.e., with a higher dose rate).

Therefore, the comparison of different types of X-rays with different energies is not easy since often they are used with different dose rates, and the latter can be a confounding factor. For example, in their study on neoplastic transformation, Frankenberg et al. [5] compared 200 and 29 kVp X-rays, but the first was delivered at a dose rate of 0.83 Gy/min and the second at 1.13 Gy/min (thus possibly overestimating the RBE of the 29 kVp X-ray).

We would like to add that although the evidence for the influence of photon energy on biological effectiveness is not so recent (see above), the characterisation of X-rays is often omitted in published articles. Indeed, searching for articles on Pubmed.gov that contain “X-ray” and “DNA damage” and “cells” in the title and/or abstract returns 1125 papers, of which only 36 contain “kV” or “kVp” or “keV”.

The aim of this study was to compare the biological effectiveness of X-rays with different photon energies (obtained with different applied peak potentials, kVp) but with the same dose rate. Secondarily, we also studied X-rays with different dose rates but with the same photon energy. Biological effectiveness was assessed by measuring induced DNA damage (mainly using the micronucleus test, but also with the comet test) and cell survival. Our results, confirming previous observations in a more sound and consistent way, underline the limitations of the dose as the sole dosimetric parameter for in vitro studies.

## 2. Results and Discussion

### 2.1. Influence of the Dose Rate on the Dose–Response Relationship

**Figure 1:**
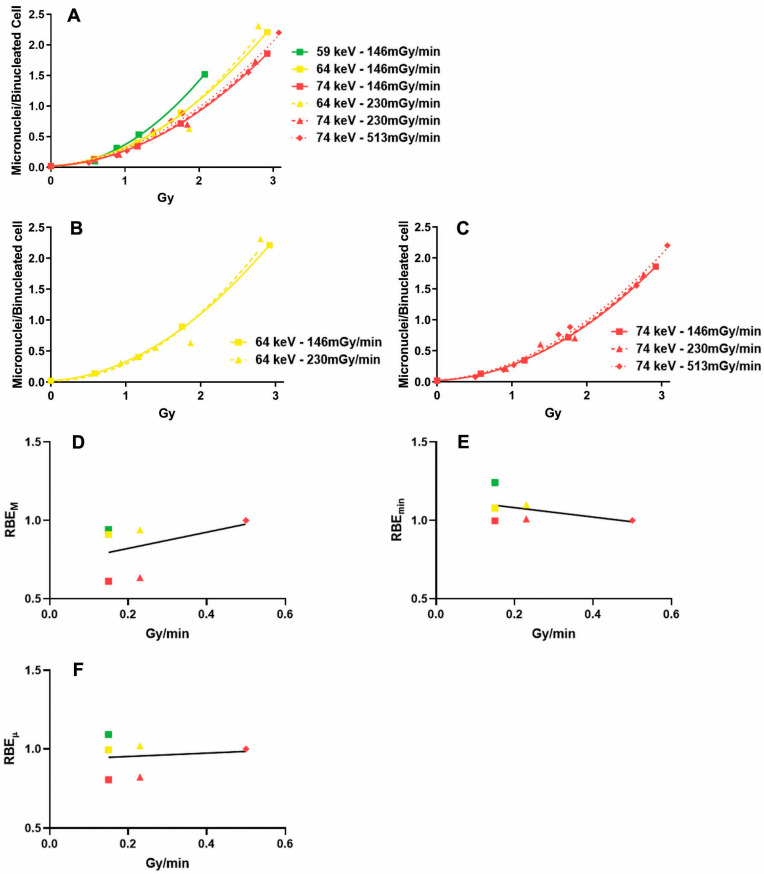
Influence of the dose rate on micronuclei induction in X-irradiated cells. (**A**) Micronuclei frequencies in samples exposed to X-rays with different dose rates and/or photon energies. (**B**) Micronuclei frequencies in samples exposed to 64 keV X-rays with different dose rates. (**C**) Micronuclei frequencies in samples exposed to 74 keV X-rays with different dose rates. (**D**) Regression between dose rate and RBE_M_. (**E**) Regression between dose-rate and RBE_min_. (**F**) Regression between dose-rate and RBE_µ_. Colors represent photon energies (green = 59 keV; yellow = 64 keV; red = 74 keV). Symbol shapes represent dose rates (squares = 146 mGy/min; traingles = 230 mGy/min; diamonds = 513 mGy/min).

Different cell lines, because of differences in DNA damage checkpoint stringency, have different yields of acentric fragments (which give place to micronuclei) for the same dose [19]. In this work, we used the SV40-transformed cell line B3, which allowed us to study micronuclei induction for doses up to 3 Gy (whereas, for example, fibroblast cultures are completely blocked at 2 Gy). Figure 1A shows the results of micronuclei frequencies in cells exposed to X-rays with different dose rates and/or photon energies.

Using a 64 keV X-ray, we found that in samples exposed to 230 mGy/min, there was a slight, not significant increase in micronuclei induction compared to cells exposed to 146 mGy/min (Figure 1B). Indeed, the difference between the two curves was not significant (ΔAICc = −25.2; probability < 0.01%). Also, with a 74 keV X-ray (Figure 1C), the differences we found in micronuclei induction between cells exposed to 146, 230, and 513 mGy/min were not significant (ΔAICc = −14.4; probability = 0.03%).

Considering all types of X-rays employed, we found that the regression between the dose rate and RBE_M_ was not significant (Figure 1D, r2 = 0.17, *p* = 0.42). Also, the regressions with the RBE_min_ (Figure 1E) and RBE_µ_ (Figure 1F) were not significant (r2 = 0.17, *p* = 0.39 and r2 = 0.02, *p* = 0.81, respectively). However, it is intuitive from these figures that these regressions are highly influenced by the differences in photon energy.

Differently from previous studies, we did not find significant variations in the dose response due to different dose rates. However, it should be noted that the differences in dose rate we could employ (ranging from 146 to 513 mGy/min) were very small if compared to the ranges employed by Ruiz de Almodovar et al. [14] (0.01–1.28 Gy/min), Dikomey and Brammer [15] (0.04–4 Gy/min), and Bertucci et al. [16] (3.1 mGy/min–1 Gy/min). Nonetheless, in the successive experiments on the biological effectiveness of X-rays with different photon energies, we employed constant dose rates in order to avoid a possible confounding factor.

### 2.2. Influence of Photon Energy on the Dose–Response Relationship

**Figure 2:**
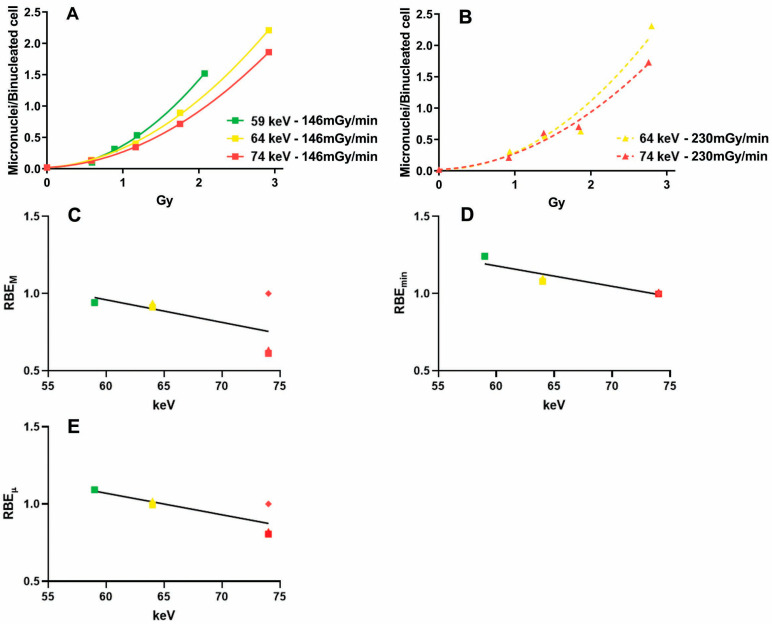
Influence of photon energy on micronuclei induction in X-irradiated cells. (**A**) Micronuclei frequencies in samples exposed to 146 mGy/min X-rays with different photon energies. (**B**) Micronuclei frequencies in samples exposed to 230 mGy/min X-rays with different photon energies. (**C**) Regression between photon energy and RBE_M_. (**D**) Regression between photon energy and RBE_min_. (**E**) Regression between photon energy and RBE_µ_. Colors represent photon energies (green = 59 keV; yellow = 64 keV; red = 74 keV). Symbol shapes represent dose rates (squares = 146 mGy/min; traingles = 230 mGy/min; diamonds = 513 mGy/min).

Employing X-rays with different energies (59, 64, and 74 keV) but with a constant dose rate of 146 mGy/min (Figure 2A), we found significant differences between the curves fitting the micronuclei induction (ΔAICc = 38.8; probability > 99.99%), with the 59 and 74 keV X-rays showing, respectively, the highest and the lowest induction of micronuclei. In the case of a constant dose rate of 230 mGy/min (Figure 2B), we found again differences between the 64 and 74 keV X-rays (ΔAICc = 1.67; probability = 69.65%).

Considering all types of X-rays employed, we found that the regression between the mean photon energy and the RBE_M_ was not significant (Figure 2C, r2 = 0.33, *p* = 0.24). On the other hand, the regressions with the RBE_min_ (Figure 2D) and RBE_µ_ (Figure 2E) were significant (r2 = 0.67, *p* = 0.0064 and r2 = 0.64, *p* = 0.05, respectively).

Since both the dose rate and mean photon energy may influence the response to X-ray exposure, we also conducted multiple regressions with these two quantities as independent variables. The regression with the RBE_M_ was significant (r2 = 0.95, *p* = 0.0128), and both the regression coefficients for the dose rate (positive) and photon energy (negative) were significantly different from zero (*p* = 0.01 and *p* = 0.007, respectively). The regression with the RBE_min_ was significant (r2 = 0.67, *p* = 0.046), but only the regression coefficient for photon energy was significantly different from zero (*p* = 0.028). Finally, the regression with the RBE_µ_ was significant (r2 = 0.99, *p* = 0.0012), and both the regression coefficients for the dose rate and photon energy were significantly different from zero (*p* = 0.0024 and *p* = 0.005, respectively).

Since we used a single cell line for our experiments, we report also some results obtained (employing the micronucleus test) in our laboratory with other cell lines (Table 1). Although dose responses differ significantly between cell lines (as described by the α and β coefficients), it can be seen that the influences of photon energy and dose rate on biological effectiveness we saw before are confirmed. In fact, the RBE_M_ and RBE_µ_ decrease with a decreasing dose rate and increase with a decreasing photon energy. Perhaps employing very radioresistant cell lines, allowing for the micronucleus test to be performed with very high doses (up to 8–10 Gy) when the quadratic component β is predominant, will give more substantial results regarding the RBE_min_.

**Figure 3:**
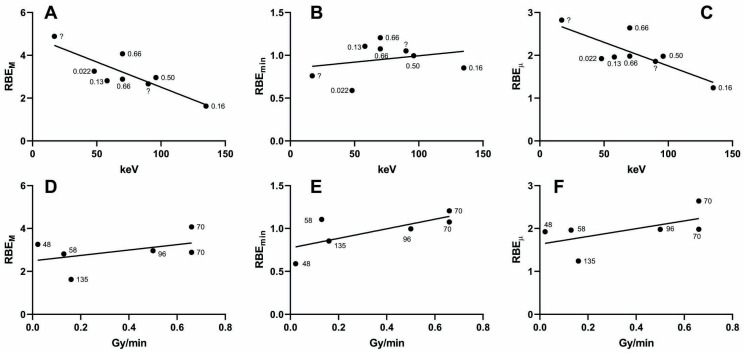
Biological effectiveness of different types of X-rays, measured by dicentric induction. (**A**) Regression between photon energy and RBE_M_. (**B**) Regression between photon energy and RBE_min_. (**C**) Regression between photon energy and RBE_µ_. (**D**) Regression between dose rate and RBE_M_. (**E**) Regression between dose rate and RBE_min_. (**F**) Regression between dose rate and RBE_µ_. The numbers next to the points denote the dose rate (in Gy/min) in (**A**–**C**) and photon energy (in keV) in (**D**–**F**). A question mark denotes an unavailable dose rate.

As stated in the Introduction, previous studies were already conducted on the variation in the RBE as a consequence of different photon energies. In particular, the most abundant data are on dicentric induction. Guerrero-Carbajal et al. [9] and Hill [10], using their own and previous results, showed an inverse relationship between the mean photon energy and the RBE_M_, but this was done by describing graph plots and without statistical analyses. We therefore reanalysed those data, considering not only photon energies but also dose rates (when available). We confirmed the inverse relationship between photon energy and the RBE_M_ (Figure 3A, r2 = 0.72, *p* = 0.0076) and we found a significant result also with the RBE_µ_ (Figure 3C, r2 = 0.63, *p* = 0.019) but not with the RBE_min_ (Figure 3B, r2 = 0.07, *p* = 0.54). Concerning the direct relationship with the dose rate, we found no significant results either with the RBE_M_ (Figure 3D, r2 = 0.21, *p* = 0.37), RBE_min_ (Figure 3E, r2 = 0.52, *p* = 0.11), or RBE_µ_ (Figure 3F, r2 = 0.34, *p* = 0.22). Performing multiple regressions with dose rate and photon energy as independent variables, we found that the regression with the RBE_M_ was significant (r2 = 0.95, *p* = 0.05) and the (negative) regression coefficient for photon energy was significantly different from zero (*p* = 0.03), while the one for dose rate was not (*p* = 0.085). The regression with the RBE_min_ was not significant (r2 = 0.46, *p* = 0.54). Finally, the regression with the RBE_µ_ was significant (r2 = 0.99, *p* = 0.0043), and both the regression coefficients for dose rate and photon energy were significantly different from zero (*p* = 0.005 and *p* = 0.003, respectively). In conclusion, our reanalyses of past data on dicentrics seem to confirm the results from our own data, i.e., that both dose rate and photon energy influence the biological effectiveness of an X-ray.

**Figure 4:**
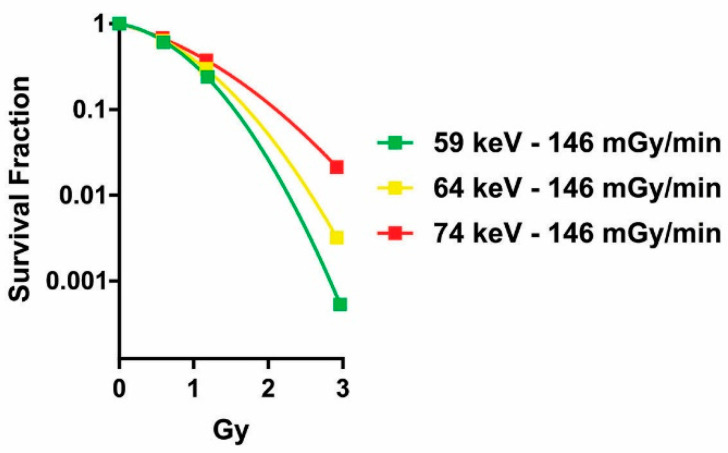
Influence of photon energy on cell survival. Results of a 14-day clonogenic assay in samples irradiated with 146 mGy/min and different photon energies.

We also looked for confirmation of the differences we found between X-rays with different energies by using another endpoint, i.e., a clonogenic assay (Figure 4). Once again, we observed that the lower the energy of the X-ray, the stronger the effect (i.e., lower survival fraction). Indeed, the differences between the curves fitting the survival fractions were significant (ΔAICc = 60.3; probability > 99.99%).

**Figure 5:**
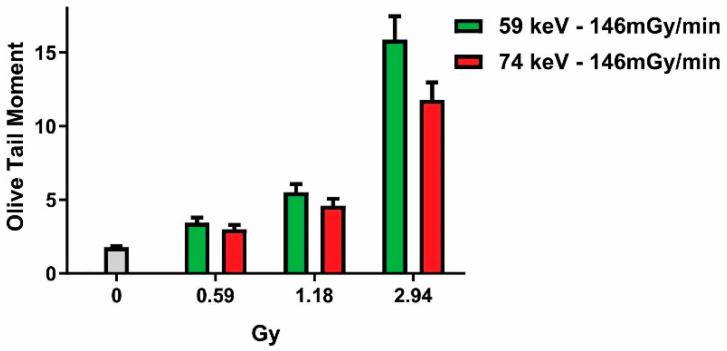
Influence of photon energy on double-strand-break induction. DNA damage evaluated as the olive tail moment via comet assay in samples irradiated with 146 mGy/min and different photon energies.

Since micronuclei are a product of unrepaired DSBs (and as survival is mainly determined by these), one could raise the question of if X-rays with different photon energies have different degrees of biological effectiveness either because they produce different levels of DSBs or because they cause the same number of DSBs, which is repaired with different efficiencies depending on the photon energy. Therefore, we measured the initial level of DSBs by performing the comet assay immediately after the end of irradiation, using X-rays with the most divergent effects (i.e., 59 vs. 74 keV, Figure 5). A paired *t*-test revealed a significant difference (*p* = 0.049) between the 59 and 74 keV X-rays (with the same dose rate), confirming that the photon energy is inversely correlated with the amount of produced DSBs.

### 2.3. Absorbed Dose Versus Fluence

**Figure 6:**
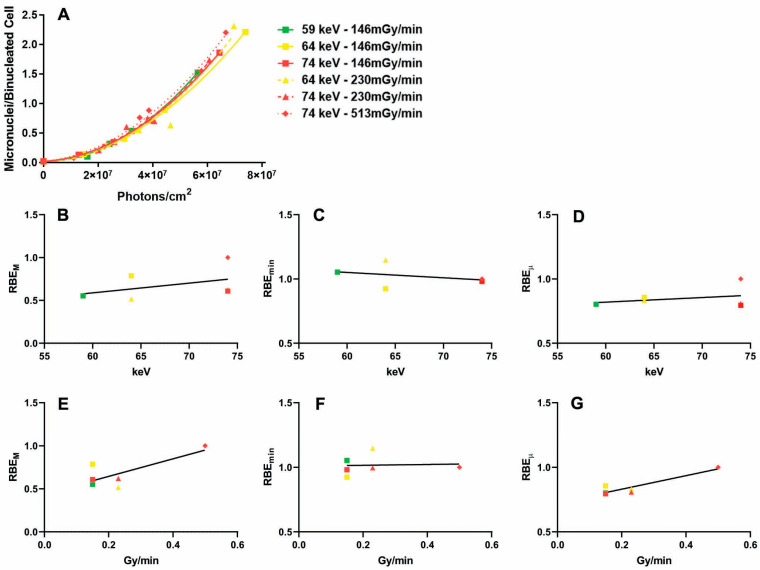
Relationship between micronuclei frequencies and incident photons. (**A**) Micronuclei frequencies in samples exposed to X-rays with different dose rates and/or photon energies; note that the independent variable is the number of photons and not the absorbed dose; (**B**) regression between photon energy and RBE_M_; (**C**) regression between photon energy and RBE_min_; (**D**) regression between photon energy and RBE_µ_; (**E**) regression between dose rate and RBE_M_; (**F**) regression between dose rate and RBE_min_; (**G**) regression between dose rate and RBE_µ_. Colors represent photon energies (green = 59 keV; yellow = 64 keV; red = 74 keV). Symbol shapes represent dose rates (squares = 146 mGy/min; traingles = 230 mGy/min; diamonds = 513 mGy/min).

Since for a given dose, cells irradiated with X-rays with lower photon energies receive a greater number of photons, we investigated the relationship between micronuclei induction and incident fluence (i.e., the number of photons reaching the exposed cells, Figure 6A). In this case, the RBE_M_, RBE_min_, and RBE_µ_ were calculated from the classical linear–quadratic equation, but the independent variable was the number of incident photons (instead of the dose).

We found that all the regressions between the mean photon energy and the RBE_M_, RBE_min_, and RBE_µ_ were not significant (Figure 6B–D, r2 = 0.17, 0.13, 0.09 and *p* = 0.41, 0.48, 0.55, respectively). On the other hand, the regressions between the dose rate and the RBE_M_ (Figure 6E) and RBE_µ_ (Figure 6G) were significant (r2 = 0.58, 0.85 and *p* = 0.05, 0.009).

Thus, our results indicate that DNA damage is determined by the number of photons hitting the cell, independent from the photon energy. On the other hand, the dose rate (which can be converted into the photon flux, i.e., the number of photons hitting the cell per second) influences biological effectiveness.

### 2.4. Incident Photons and DSBs

As stated in the Introduction, the fact that soft X-rays have more marked biological effects than hard X-rays has been known for a century [12,13]. Although representing a very small portion of radiobiological studies, several articles reported comparisons of DNA damage induced by X-rays with different photon energies. Using data on dicentrics (the most used endpoint) obtained by several researchers, some authors [9,10] showed an inverse relationship (although through graph plots without statistical analyses) between the mean photon energy and RBE_M_. Nonetheless, the differences in biological effectiveness among 50–100 keV X-rays are usually considered absent or negligible. In the present study, we observed that differences in the RBE in the 59–74 keV range were detectable after controlling for dose-rate variations. In addition, our reanalysis of previous data may indicate that the perceived lack of differences in the RBE among 50–100 keV X-rays is perhaps due to the different dose rates employed.

The biophysical mechanism determining the inverse relationship between photon energy and the RBE has already been proposed [22]. X-ray-induced DSBs are mainly ascribable to the effects of secondary electrons (produced by the interactions of X-ray photons with water molecules in the cell). For photon energies below 50 keV, the photoelectric effect is predominant, while at higher energies, the Compton effect dominates (and thus not all the energy is transferred to electrons). Therefore, the shift from the photoelectric to the Compton effect determines a decrease in the mean electron energy with an increasing photon energy. Also particularly important are Auger electrons (which are 0.5 keV in water) as another source of DSBs. Indeed, they accompany all photoelectrons but only 20% of Compton effect electrons. Considering these mechanisms, differences in the RBE in the 50–100 keV range are believed to be minimal. Nonetheless, secondary electrons are the main, but not the sole, source of DNA damage. Another source comprises ^•^OH radicals, which induce single-strand breaks (a fraction of which, independently of their origin, can give place to DSBs if close enough one to another). Since for electrons with energy > 1 keV, the initial ^•^OH radical yield is independent of the electron energy [23], it can be expected that radical production is proportional to the number of incident photons. Thus, since for a given dose, X-rays with lower photon energies have more photons, they will produce more ^•^OH radicals than the same X-ray dose with a higher photon energy. Of course, this speculation needs to be tested experimentally. Another issue that deserves further study is the production of 8-oxo-dG, a DNA lesion that is processed into a DSB during DNA replication and for which (as far as we know) there are no studies exploring its relationship with photon energies and dose rates.

## 3. Materials and Methods

### 3.1. X-Irradiation

Different types of X-rays were produced using dedicated Gilardoni (Gilardoni, Italy) radiological equipment (6 mA; 0.3 mm Cu filter), applying 100, 120, and 168 kVp peak potentials (producing 58.9, 64.3, and 73.8 keV mean photon energies). With a fixed kVp, different dose rates were obtained by placing the samples at different distances (ranging 14–44 cm) from the radiogenic tube. In this way, we obtained X-rays with different photon energies but with the same dose rate and, vice versa, X-rays with different dose rates but with the same photon energy (Table 2). Spectra and dosimetry were conducted as reported in a previous work [24].

### 3.2. Cell Cultures

Human Lens Epithelial Cells B3 (ATCC, Manassas, VA, USA) were grown in Eagle’s minimal essential medium with Earle’s salt (Euroclone, Milano, Italy) supplemented with 20% foetal bovine serum (Euroclone, Italy), with 10,000 units/mL of penicillin, 10 mg/mL of streptomycin (Biological Industries, Beit-Haemek, Israel), and 1% non-essential amino acid (Euroclone, Italy). The cells were grown in an incubator at 37 °C, with 95% relative humidity and 5% CO_2_.

This cell line was chosen because following treatment with Cytochalasin-B (see Section 3.3), binucleated cells can be obtained with doses up to ≈3 Gy, whereas other cell lines (e.g., fibroblasts) are completely blocked at 2 Gy, showing no binucleated cells and thus making the micronucleus test unfeasible [19].

### 3.3. Micronucleus Test

One day before irradiation, 50,000 cells were seeded inside 35 mm Petri dishes. Cytochalasin-B (final concentration: 3 μg/mL in DMSO, Sigma-Aldrich, Burlington, MA, USA) was added immediately after irradiation. After 24 h, slides were fixed with methanol and stained with 1 μg/mL of DAPI (4′,6-diamidino-2-phenylindole, Sigma-Aldrich, USA). Micronuclei (MN) were scored at 63× magnification using a Zeiss (Carl Zeiss, Oberkochen, Germany) microscope with ultraviolet light (359 nm excitation filter; 441 nm barrier filter). MN frequencies were assessed scoring 1000 binucleated cells (BNC) for each sample. For each experimental point, at least 3 independent replicates were used.

### 3.4. Clonogenic Assay

One day before irradiation, cells were seeded inside 35 mm Petri dishes. After X-irradiation, the cells were incubated (without changing medium) for 14 days, then fixed with methanol and stained with gentian violet. Colonies with >50 cells were scored for cell surviving fraction determination. The surviving fraction at each dose was calculated relative to the plating efficiency of non-irradiated control cells.

### 3.5. Comet Assay

The neutral comet assay (which allows for the detection of double-strand breaks (DSBs)) was performed as in [25] with minor modifications. One day before irradiation, 50,000 cells were seeded inside 35 mm Petri dishes. In order to prevent DNA repair during X-ray exposure, irradiation was performed on ice. At the end of X-irradiation, the cells were immediately trypsinised, harvested, and centrifuged at 1200 rpm for 8 min; the pellet was then resuspended in a phosphate-buffered saline (PBS) solution and mixed with 0.7% low melting agarose. This suspension was then spread on a slide precoated with normal melting agarose and covered with a coverslip. After removing the coverslips, the slides were left submerged in a cold lysis solution (100 mM Na2EDTA, 2.5 M NaCl, and 10 mM Tris) overnight and then placed in the electrophoresis chamber in the dark with a TBE buffer solution (2 mM Na2EDTA, 90 mM Tris, 90 mM boric acid; pH, 8.0). After DNA denaturation (20 min at +4 °C) and electrophoresis (25 V, 300 mA, 20 min), the slides were washed in 0.4 M Tris at a pH of 7.5, fixed in cold absolute ethanol and air dried. Immediately before analysis, the slides were stained with ethidium bromide (10 μL/ml), and images were then acquired using an Axio Imager M1 fluorescent microscope (Carl Zeiss) equipped with a CCD camera. For each sample, 100 images were analysed using Comet Score software version 2.0 (developed by Rex Hoover, Sumerduck, VA, USA), evaluating the olive tail moment as a normalised measure of DNA damage. For each experimental point, at least 3 independent replicates were used. 

### 3.6. Statistical Analyses

Dose–response relationships were studied, calculating regression coefficients with the ordinary least squares approach. For the dose–micronuclei relationship, the regressions were modelled as $y=\alpha x+\beta x^{2}+c$, while for the dose–surviving fraction relationship, the regressions were modelled as $y=e^{-\left(\alpha x+\beta x^{2}\right)}$ (using a linear–quadratic model in both cases). In order to study if the regression curves of different types of X-rays are significantly different, differences in the Akaike corrected information criterion (ΔAICc) were calculated, reporting also the probability (calculated as $\frac{e^{0.5\Delta }}{1+e^{0.5\Delta }}$) that the model is correct. 

Since the RBE changes with the dose, in order to compare more than two types of X-rays and to analyse regressions, we calculated the widely used “maximum RBE” as ${RBE}_{M}=\alpha_{n}/\alpha_{r}$, where *α_n_* is the linear coefficient of the investigated radiation and *α_r_* is the one of the reference radiation [26]. As a ratio of the linear coefficients of the linear–quadratic equation, it reflects the situation at low doses (when the quadratic component is negligible). We also calculated the “minimum RBE” as ${RBE}_{min}=\sqrt{\beta_{n}/\beta_{r}}$ [27] which, in turn, reflects the situation at high doses (when the quadratic component is predominant). We also used the mean between the RBE_M_ and RBE_min_, which we defined as the RBE_µ_, a quantity that incorporates both characteristics of the radiation under consideration.

Between our types of X-rays, we took the one with 74 keV and 0.51 Gy/min as reference.

Simple and multiple regressions were calculated using the ordinary least squares method.

The results of the comet assay were compared using a paired-samples *t*-test.

For all analyses, the level of significance was set at *p* < 0.05.

## 4. Conclusions

The fact that different types of X-rays have different degrees of biological effectiveness is known but seems usually to be underrated. In some articles (especially those focused more on genotoxicity per se than on radiobiology), different studies with “X-ray” are compared irrespective of photon energies and/or dose rates. Moreover, many authors compare the RBE they find for a type of radiation (heavy ions, protons, etc.) with the ones found by other researchers without checking if the reference radiation is effectively the same, leading to uncertainty and/or conflicting results. For example, the RBE for tritium (as calculated with the same endpoint and with the same type of cells) has been found to vary between 1.9 and 8 (as reviewed in [28]). Also, in the case of mammographic X-rays (25–30 kVp), reported RBE values vary from 1.1 to 4.8 [29], with important consequences on the establishment of safety standards. Indeed, the lack of an international recommendation for the definition of a specific photon source as reference radiation has been underlined [30]. Although the first original reference radiation for the weighting factor in radiation protection was quite specific (“γ radiation from radium filtered by 0.5 mm of platinum”, ref. [31]), it was later abandoned. We would suggest adopting a 200 kV, 0.5 Cu filtered X-ray at a dose rate of 1 Gy/min as standardised reference radiation. However, while this would be feasible for in vitro studies (like ours), it is impractical for in vivo, long-term studies. Nonetheless, if the γ-ray will be adopted as reference radiation, we think that this should be accompanied by precise specifications (e.g., ^60^Co, filtering, dose rate).

While for in vivo studies the absorbed dose is essential (since the biological effect in a tissue/body is the sum of the effects in different layers), for in vitro research, the dose alone is an insufficient measure. In line with our point of view, awareness about the need for accurate dose reporting in radiobiological studies is leading to the idea that scientific journals should strengthen their minimal dosimetry requirements [32,33].

In conclusion, in addition to the issues we demonstrated that deserve further investigation, we suggest considering photon energy an important characterising parameter that should not be omitted in studies on X-rays (just as the linear energy transfer for heavy ions, protons, etc., is always reported). Therefore, we suggest to every researcher using X-rays to report (in addition to the doses employed) nature the of the anode, the peak potential (kVp), filtering, and the mean photon energy (which can be easily calculated using many types of software), as well as the dose rate.

## Figures and Tables

**Table 1:** Biological effectiveness of different types of X-rays employing the micronucleus test in different cell lines.

Cell Line ^1^	Photon Energy (keV)	Dose Rate (Gy/min)	α	β	RBE_M_ ^2^	RBE_min_ ^2^	RBE_µ_ ^2^	Reference
B3	**74**	**0.51**	**0.085**	**0.199**	**1**	**1**	**1**	This study
B3	74	0.23	0.054	0.202	0.64	1.01	0.82	This study
B3	64	0.23	0.08	0.240	0.94	1.10	1.02	This study
HFFF2	**74**	**0.51**	**0.061**	**0.017**	**1**	**1**	**1**	[19]
HFFF2	74	0.23	0.043	0.016	0.7	0.99	0.84	This study
HFFF2	64	0.23	0.056	0.02	0.92	1.09	1.01	This study
Cl-1	**74**	**0.7**	**0.05**	**0.022**	**1**	**1**	**1**	[20]
Cl-1	68	0.7	0.056	0.025	1.12	1.06	1.09	[21]

^1^ B3—SV40-transformed human lens epithelial cells; HFFF2—normal human foreskin fibroblasts; Cl-1—Chinese hamster embryonic lung cells. ^2^ For each cell line, RBE_M_, RBE_min_, and RBE_µ_ are calculated taking the first X-ray type (in bold) as a reference.

**Table 2:** Characteristics of the different types of X-rays employed.

**Dose Rate**	**Peak Potential**		**Peak Potential**	**Dose Rate**
146 mGy/min	100 kVp		100 kVp	146 mGy/min
	120 kVp		120 kVp	146 mGy/min
	168 kVp			230 mGy/min
230 mGy/min	120 kVp		168 kVp	146 mGy/min
	168 kVp			230 mGy/min
513 mGy/min	168 kVp			513 mGy/min

## Data Availability

All data are available in the main text.
